# An Insight into the Role of Marine Biopolymer Alginate in Endodontics: A Review

**DOI:** 10.3390/md20080539

**Published:** 2022-08-22

**Authors:** Galvin Sim Siang Lin, Chia Yee Cher, Yong Hong Goh, Daryl Zhun Kit Chan, Mohmed Isaqali Karobari, Josephine Chang Hui Lai, Tahir Yusuf Noorani

**Affiliations:** 1 Department of Dental Materials, Faculty of Dentistry, Asian Institute of Medicine, Science and Technology (AIMST) University, Bedong 08100, Kedah, Malaysia; 2 Conservative Dentistry Unit, School of Dental Sciences, Health Campus, Universiti Sains Malaysia, Kubang Kerian, Kota Bharu 16150, Kelantan, Malaysia; 3 Centre for Multidisciplinary Research (CFTR), Saveetha Dental College, Saveetha Institute of Medical and Technical Sciences, Saveetha University, Chennai 600077, India; 4 Department of Restorative Dentistry & Endodontics, Faculty of Dentistry, University of Puthisastra, Phnom Penh 12211, Cambodia; 5 Department of Chemical Engineering and Energy Sustainability, Faculty of Engineering, Universiti Malaysia Sarawak, Kota Samarahan 94300, Sarawak, Malaysia

**Keywords:** alginate, biomaterial, biopolymer, drug delivery system, hydrogel, polysaccharide, regenerative dentistry

## Abstract

Alginate is a natural marine biopolymer that has been widely used in biomedical applications, but research on its use as an endodontic material is still sparse in the literature. This pioneer review aims to summarize the emerging roles of alginate and to outline its prospective applications as a core biomaterial in endodontics. Ten electronic databases and five textbooks were used to perform a search of English-language literature on the use of alginate in endodontics published between January 1980 and June 2022. The risk of bias (RoB) of each included study was assessed using the Office of Health Assessment and Translation (OHAT) tool. Subsequently, studies were categorized into three tiers to represent the overall risk. Qualitative analysis was performed, and the articles were sorted into different thematic categories. An initial search yielded a total of 1491 articles, but only 13 articles were chosen. For most domains, all the studies were rated with ‘probably low’ or ‘definitely low’ RoB, except for domains 2 and 6. All included studies fall in the Tier 1 category and were either in vitro, in vivo, or ex vivo. Four thematic categories were identified: endodontic regeneration, intracanal medicament, filing material, and chelating agent. Based on the available evidence, alginate has emerged as a cell carrier and scaffold in regenerative endodontics, a microcapsule delivery system for intracanal medicaments, a chelating agent reinforcing material, and a root canal sealer. More well-designed experiments and clinical trials are needed to warrant the promising advent of this hydrogel-based biomaterial.

## 1. Introduction

A biomaterial is any natural or synthetic material that makes up all or part of a living structure or biomedical device that performs, enhances, or substitutes a natural function [1]. Biomaterials have historically been meant to be inert and do not interact with the host’s biological processes [2]. Natural resources have a long history as biomaterials and have been used to replace tissues lost due to disease or injury [3]. Dental biomaterials are natural tissues and biocompatible synthetic materials that can restore decayed, injured, or fractured tooth tissue. In restorative dentistry, biomaterial research has evolved towards regenerative (resorbable) or bioinert (biostable) materials to enhance material adherence, promote faster healing, and enable rapid tissue regeneration [4,5,6].

Alginate or salt of alginic acid is one of the most abundant biopolymeric hydrocolloids derived primarily from seaweeds [2,7]. Alginate constitutes blocks of (1–4)-linked β-D-mannuronic acid (M) and α-L-guluronic acid (G) monomers that are composed of three different polymer segments [8]. It can form hydrogel by participating in intermolecular cross-linking with divalent cations such as calcium ion (Ca^2+^). In addition, alginate has piqued the interest of many researchers because of its biocompatibility, low cost, acceptable smell and taste, simplicity of handling, low toxicity, and mild gelation properties [9]. The structural resemblance of alginate hydrogel to the extracellular matrix of living tissues allows for a wide range of applications of this bio-polysaccharide in wound healing, cell transplantation, and tissue engineering or regeneration [2]. Another benefit of alginate is its capability to hydrate and form gel, allowing specific medicaments or active substances to be administered for longer periods at the site of interest, thus acting as controlled-release drug delivery systems [2,4,7].

In dentistry, hydrocolloid alginate has been commonly employed as a dental impression material to make gypsum casts for various treatment purposes, including provisional crowns and bridges, orthodontic study models, mouth guards, bleaching trays, and removable dental prostheses [7,9]. Endodontics is a discipline of dentistry that focuses on the diagnosis and treatment of inflamed and infected dental pulp tissues [10]. Undoubtedly, dental caries or tooth decay continues to be a key contributing factor to the need for endodontic treatment [11]. Along with the declining rate of tooth loss, this highlights the significance of continuously improving biomaterials used in endodontic applications, such as pulp therapies, irrigating solution, intracanal medicaments, obturation, and pulpal regeneration [11]. Moreover, researchers have been intrigued by incorporating alginate into the field of endodontics due to its remarkable features [4,12]. For instance, alginate has been introduced as a regenerative scaffold because of its semi-permeable characteristic that allows cells to adhere, proliferate, and differentiate [13,14]. The sol–gel transition ability of alginate as endodontic biomaterial also permits it to be fabricated as preformed or injectable scaffolds that match the root canal system’s complicated architecture [5].

Even though alginate is now widely used as a dental material, research on its specific usage as endodontic material is still sparse in the literature. Hence, the objective of this pioneer review was to identify and summarise the contemporary roles of alginate biopolymer in endodontics and to sketch out prospective future research on the application of alginate as the core endodontic biomaterial.

## 2. Materials and Methods

This review was conducted to identify, summarise, and map the role of alginate in the field of endodontics. The inclusion criteria for study selection were: (1) application of alginate in endodontics; (2) randomised, non-randomised, cohort, case–control, cross-sectional, in vitro, in vivo, ex vivo studies; (3) article published in English language. The exclusion criteria were: (1) application of alginate in other dental specialities, such as prosthodontics or periodontics; (2) reviews, expert opinions, letters to the editor, case reports, or case series.

### 2.1. Study Selection

Ten electronic databases (PubMed, Cochrane, LILACS, Science Direct, Web of Science, EMBASE, OpenGrey, EBSCO, Google Scholar, Library Genesis) were searched independently by four investigators (G.S.S.L., C.Y.C., D.Z.K.C., Y.H.G.) using a combination of keywords: ‘algin’, ‘alginate’, ‘alginic acid’, ‘hydrogel’, ‘hydrogels’, ‘endodontics’, ‘endodontic’, ‘root canal’, and ‘root canals’. The Boolean operators ‘AND’ and ‘OR’ were employed during the search and the screening procedure covered articles published from January 1980 to June 2022. In addition, relevant studies from five textbooks were hand-searched [4,15,16,17,18]. The reference lists of selected articles were further checked to identify studies that may be eligible for the present review. 

### 2.2. Data Extraction 

After an initial search, duplicated articles were excluded using EndNote software (version x9, Thomson Reuters, Phila, PA, USA), and the remaining articles were screened independently based on the title and the abstract by two investigators (T.Y.N., J.C.H.L.). Four other investigators (G.S.S.L., C.Y.C., D.Z.K.C., Y.H.G.) subsequently conducted a full-text evaluation to select eligible studies based on the inclusion and exclusion criteria. Calibrations between investigators were performed to assess interrater reliability. To compare the investigators’ decisions on inclusion and exclusion, the average concordance was calculated using the Kappa value [19]. With the assistance of the seventh investigator (M.I.K.), any conflicts that developed throughout the search were addressed and resolved.

Next, three investigators (C.Y.C., D.Z.K.C., Y.H.G.) independently extracted data using a custom-prepared data extraction form, while another investigator (G.S.S.L.) validated the accuracy of the retrieved data. The following study characteristics were collected from each included article: authors, year of publication, country, study design, and general outcome. Qualitative thematic analysis was performed by two investigators (C.Y.C., D.Z.K.C.) through identifying and classifying emerging themes of alginate application in the field of endodontics. Researcher triangulation was employed with the third investigator (G.S.S.L.) to validate the thematic contents. 

### 2.3. Risk of Bias Assessment

The risk of bias (RoB) for each included study was assessed using the Office of Health Assessment and Translation (OHAT) Risk of Bias Assessment Tool from the National Toxicology Programme (NTP) [20]. The OHAT assessment tool was also modified to account for in vitro, in vivo, and ex vivo experimental study designs. A list of ten domains was used to identify potential bias, as well as a supplementary category for ‘other potential threats to internal validity’. Moreover, only questions 1, 2, 5, 6, 7, 8, 9, 10, and 11 were applied to evaluate experimental studies. 

The 11th question, labelled ‘other bias’ by OHAT, allows for the incorporation of other possible risks to internal validity (e.g., statistical methods). Each RoB question was addressed on a four-point scale: ‘definitely high’, ‘probably high’, ‘probably low’, and ‘definitely low’. ’NR’ was assigned when insufficient information was retrieved or not reported from the selected study. The assessments were completed independently by two investigators (C.Y.C., D.Z.K.C.), and any discrepancies were resolved through discussion with the third investigator (G.S.S.L.).

Following the assessment of internal validity using the OHAT risk of bias tool, studies were categorised into tiers to represent the overall risk of bias. According to the OHAT guidance, a three-tier method was used [20]. The key domains indicated for in vitro, in vivo, and ex vivo were comparable due to the nature of experimental study designs, with domains 5 and 9 being identified as key [21]. 

Tier 1: A study’s key domains must be rated ‘definitely low’ or ‘probably low’ with most other domains rated ‘definitely low’ or ‘probably low’ (greater degree of confidence).

Tier 2: A study that does not fulfil the tier 1 or tier 3 criteria.

Tier 3: A study’s key domains must be rated ‘definitely high’ or ‘probably high’ with most other domains rated ‘definitely high’ or ‘probably high’ (lower degree of confidence).

## 3. Results

**Figure 1:**
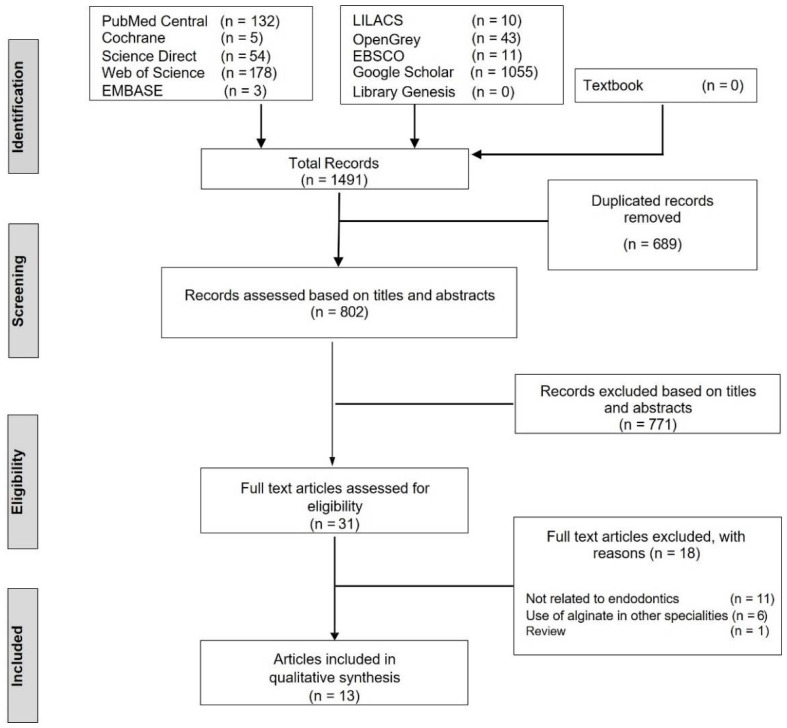
Flowchart for study selection process.

During the initial search, a total of 1491 articles were identified, of which 689 duplicate articles were eliminated, followed by the exclusion of another 771 articles after a preliminary screening based on the title and abstract. An in-depth, full-text examination of the remaining 31 articles was performed, and 13 articles were finally selected for qualitative analysis [5,6,12,13,14,22,23,24,25,26,27,28,29]. Figure 1 depicts the flow of study selection and reasons for excluding the articles, whereas Table 1 summarises the characteristics of the included studies. The preliminary article screening (titles and abstracts) average k coefficient value was 0.87, and the second article screening (full-text assessment) average k coefficient value was 0.77, signifying ‘very strong’ and ‘strong’ agreement, respectively. 

### 3.1. Study Characteristics

**Figure 2:**
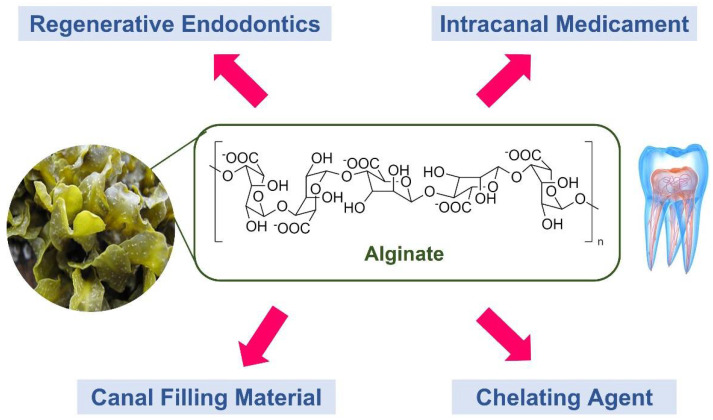
Role of alginate: endodontic regeneration, intracanal medicament, root filling material, and chelating agent.

All included articles were published within the past ten years except for one published in the year 2005 [25]. The included articles were either in vitro, in vivo, or ex vivo experimental studies, with the bulk of the research derived from Asiatic countries [5,6,12,22,23,24,27,28,29]. Four themes were categorised: endodontic regeneration, intracanal medicament, root filling material, and chelating agent (Figure 2). 

### 3.2. Risk of Bias Assessment of Selected Studies

All selected studies demonstrated either ‘probably low’ or ‘definitely low’ risk for domains 5, 7, 8, 9, and 10, while ‘definitely low’ risk was found in all included studies for domain 11 (Table 2). Conversely, all included studies showed either ‘probably high’ or ‘definitely high’ risk of bias for both domains 2 and 6. As for domain 1, all included studies were deemed to have either ‘probably high’ or ‘definitely high’ risk, except three studies that had a ‘probably low’ risk of bias [22,24,25]. Overall, all included studies fall in the Tier 1 category.

### 3.3. Qualitative Thematic Analysis

#### 3.3.1. Role of Alginate in Regenerative Endodontics

Bhoj M et al. [5] produced an alginate-based microenvironment that mimics the shape of gutta-percha and expresses the internal cellular and molecular conditions necessary for pulp tissue regeneration. Arginine–glycine–aspartate (RGD) alginate was used to encapsulate human umbilical vascular endothelial cells and dental pulp stem cells with retained growth factors. The study noted an increased proliferation of dental pulp stem cells and human umbilical vein endothelial cells signifying an excellent regenerative effect. Similarly, Devillard R et al. [13] created a biological scaffold made of alginate–collagen to serve as a biological gutta-percha for regenerative endodontic treatment. Human apical papilla stem cells were able to disperse, survive, proliferate, and differentiate into osteoblast-like cells with calcified osseous extracellular matrix on this alginate–collagen scaffold, providing an excellent root canal healing environment.

Alginate is often used as a component in many 3D-printed scaffolds due to its quick gelation characteristics and good mixing properties with other biopolymers. To encapsulate stem cells from the apical papilla, Athirasala A et al. [14] developed a hybrid 3D bio-ink hydrogel containing 3% *w*/*v* alginate that resulted in an upward tendency of cell survival over time. Such a finding corroborates with that of Yu H et al. [12], who revealed that 3D-printed alginate–gelatine scaffolds show increased cell growth and adhesion while also containing more calcium and phosphorus ions, which are favourable to cell proliferation. Moreover, Zhang R et al. [22] developed a new injectable hydrogel microsphere that can encapsulate human dental pulp stem cells and vascular endothelial growth factor by combining an arginine–glycine–aspartate–alginate (RGD-alginate) solution with different concentrations of laponite. Pure alginate was found to demonstrate the least degree of degradation with cell survival rates in RGD-alginate microspheres exceeding 90%. A similar study was conducted by Liang et al. [28] in fabricating gelatine methacryloyl–alginate core-shell microcapsules for endodontic regeneration that co-encapsulate human dental pulp stem cells and human umbilical vein endothelial cells. The results indicated that human umbilical vein endothelial cells and human dental pulp stem cells microcapsules showed greater cell proliferation rates with the formation of capillary- and odontogenic-like networks, respectively. Additionally, pre-vascularized microtissues developed in these microcapsules, containing a high content of extracellular matrix deposition. In vivo experiments also revealed that pulp-like tissue regeneration and improved microvessel creation occurred in these microcapsules.

Nonetheless, these findings contradict those of Matsumoto N et al. [24], suggesting that biomarkers transforming growth factor (TGF-1) and bone morphogenic protein-2 (BMP-2) rose significantly with remarkable healing responses when in vivo rats with apical periodontitis were treated with Emdogain (enamel matrix derivative) as compared to Propylene Glycol Alginate (PGA), a marine hydrogel-based polysaccharide. Meanwhile, Lambricht L et al. [26] examined the viability and metabolic activity of apical papilla stem cells with various compositions of alginate and commercial hyaluronic-based hydrogels. Although the overall number of cells in alginate hydrogels remained consistent, the number of living cells decreased, with hyaluronic-based hydrogels demonstrating the highest number of cells that survived. On the other hand, researchers have also attempted to use alginate-based material as cell blocks for tooth dentine regeneration [29]. Lai WY et al. [29] developed cell blocks that are made of stem cells from human dental pulp loaded with alginate/fish gelatine hydrogels as the core, along with human umbilical vascular endothelial cells loaded with silicone ion-infused fish gelatine methacrylate as the cell block’s periphery. The findings confirmed that the capacity to release Si ions enhanced mimicking of the environment, increased expressions and secretions of angiogenesis-related markers, and boosted expression of odontogenic-related markers, making it appropriate for endodontic regeneration.

#### 3.3.2. Role of Alginate as Intracanal Medicament Carrier

Chlorhexidine (CHX) is a well-known chemical substance that is commonly used as a root canal irrigating agent or intracanal medicament during endodontic therapy to disinfect and clean the root canal system. Evelyna A et al. [23] produced nanocellulose–alginate nanocomposites to encapsulate CHX and discovered that the CHX release rate was considerably greater in the infected root canal environment with the aid of these nanocomposites. Nurdin D et al. [24] carried out a similar work in which they synthesised silica microcapsules to encapsulate 2% CHX and then coated them with chitosan and sodium alginate. The study revealed that the microcapsules and CHX formed a bond that facilitated the release of CHX with the highest value noted at a pH of 5.5.

#### 3.3.3. Role of Alginate as Root Canal Filling Material

One of the cornerstones of effective endodontic treatment is to seal the root canal system with bioinert gutta-percha and sealer. Huang G et al. [6] reported that incorporating 1% sodium alginate into the liquid component of a novel bioactive glass-based root canal sealer resulted in satisfactory flowability, thickness, setting time, solubility, and a uniform morphology that is indicative of clinical endodontic usage. It also demonstrated acceptable sealing ability, as well as minimal cytotoxicity and excellent biocompatibility. 

#### 3.3.4. Role of Alginate as Reinforcement Material in a Chelating Agent

Chelating agents are used in endodontic procedures to help prepare calcified and narrow root canals by chemically softening the root canal dentinal structures and dissolving the smear layer. Girard S et al. [25] developed a new experimental root canal chelating agent that included 2% alginate, 3% aerosol, 10% Tween 80, and 18% Heme binding protein (HEBP) and compared it with commercialised chelating agents. The experimental chelating agent with alginate reinforcement had no effect on free residual chlorine in the hypochlorite solution and showed improved chelating properties with a considerable reduction in smear layers in the coronal and middle root sections.

## 4. Discussion

The current review has mapped the usage of alginate in endodontics and offered an overview of the existing evidence as well as a range of research themes that may lead to a better understanding of this marine hydrogel-based biomaterial. Undeniably, root canal or endodontic treatment is currently the standard therapy for irreversible pulpitis, but complications such as bacterial microleakage, low fracture resistance, and possible loss of vitality render the prognosis for root canal treatment dubious [30,31,32]. Thus, dental pulp stem cell regeneration has revolutionised the potential to regenerate damaged tooth dentine and pulp tissue. Moreover, a new approach has been discovered by using hydrogels to transport progenitor stem cells from the apical papilla directly into the root canal [13]. It has also been suggested that the introduction of alginate has become a growing trend, which has aided in advancing stem cell-based endodontic regeneration research and clinical applications [13,22]. 

Cell differentiation and tissue regeneration have long been connected to the rigidity of stem cell scaffold materials. Extracellular matrix scaffolds with a high rigidity allow stem cells to differentiate into odontoblasts and create mineralised tissue, whereas scaffolds with a low rigidity allow soft pulp-like tissue to regenerate more readily [33]. The currently available evidence showed that alginate has a controlled stiffness that offers promising cell survival and differentiation in endodontic engineering applications [14,22,26,29]. Alginate-based hydrogels’ mechanical characteristics and degradation profile may be enhanced with the increase in silicone content, which aids in greater Si-OH bonding [29]. Despite previous research reporting that PGA reduced cell expression of TGF-1 and BMP-2 [24], alginate continues to emerge as a potential endodontic biomaterial [2,7,14]. Nevertheless, hydrogel marine biopolymers demonstrate various advantages for stem cell proliferation, including high surface areas, the capacity for rapid material transfer, three-dimensional spaces, and extracellular matrix-like structures [22,29].

The discovery of appropriate and effective cell delivery systems is highly recommended for tissue regeneration, and one critical consideration is the ability of cell adhesion to the systems. Alginate itself does not encourage cellular adhesion and attachment in addition to having a sluggish and uncontrolled rate of degradation since it cannot be metabolised by mammals’ enzyme systems [29]. The decreased cell adherence found in alginate hydrogel may explain the lower cell viability [26]. Another explanation could be due to the absence of alginate receptors and net negative charge in mammalian cells, which hinders protein adsorption and decreases cellular adhesion [2]. Nonetheless, previous studies have shown that adopting a collagen–alginate composite hydrogel can improve the mechanical properties of the scaffold while maintaining biocompatibility and cell proliferation [13,34]. Hence, it can be anticipated that future studies may benefit from combining alginate with a hyaluronic acid hydrogel to increase cell survival in regenerative endodontics [26]. On the bright side, drug delivery microcapsules made of this biomaterial may easily be cultivated in standard cell culture plates since alginate hydrogels lack the capacity of cell adherence, which prevents cell-laden microcapsule aggregation [28]. Notwithstanding, future research should explore ways to shorten the time required for fabrication processes and microsphere delivery systems that tend to aggregate during culture.

Researchers have sought to replace gutta-percha cone with biopolymers derived from alginate to enhance cell sprouting and migration with desired adaptation to the root canal wall [5,13]. Three-dimensional bioprinting has been claimed to support this breakthrough by allowing the accurate insertion of cellularized alginate scaffolds in root canals [12,14]. To achieve a safe attachment and spatial dispersion, all dental stem cells require a 3D framework, as well as the capacity to be transferred to the proper regeneration site [5,12]. However, such findings may not be relevant in clinical settings due to inconsistencies with current clinical practice standards. Thus, future advancements in 3D biomaterial printing of dental and dental pulp tissues should spur innovative clinical regeneration and dental tissue engineering techniques. Several primary studies included in the present review employed stem cells of the apical papilla (SCAP) as the cell of interest [13,14,26]. However, they are limited in quantity and may not be available to obtain from the elderly. Thus, other mesenchymal stem cell sources, such as dental pulp stem cells (DPSCs), stem cells of human exfoliated deciduous teeth (SHED), dental follicle progenitor cells (DFPC), and bone marrow-derived mesenchymal stem cells (BMMSC), all merit further investigation, particularly for their viability in alginate-derived biomaterials. 

Due to the extreme intricate architecture of the root canal systems and the capacity of the root dentine buffering, prolonged administration of antimicrobial agents to the infected region is still restricted. Nanocellulose–alginate microcapsules are small enough to penetrate within dentin tubules and have been reported to demonstrate good stability with prolonged degradation, making them ideal as a root canal irrigating solution carrier [23,27]. However, the available previous studies only tested with 2% CHX, and therefore, future research should focus on the efficacy of alginate-based microcapsules as a carrier of other intracanal medicaments, such as calcium hydroxide, antibiotics, and bioactive and natural antibacterial components. Huang G et al. [6] developed a new alginate-incorporated bioceramic root canal sealer with acceptable physical properties and outstanding sealing ability, making it favourable for clinical usage. Hence, future research should emphasise their dislodgement resistance on root dentinal walls and their performance in vivo or ex vivo to validate their clinical relevance.

The usage of alginate in endodontics is still considered in its infancy stages as several drawbacks remain unsolved. Future studies should explore ways to regulate the porosity of alginate hydrogel to promote cellular proliferation and transport, as well as to supply sufficient nutrients and prevent the death of microencapsulated cells in the alginate hydrogel scaffold [35]. Since alginate itself does not encourage cellular adhesion and attachment, more research on this aspect is warranted. It is also still unclear how to implement dynamic and intelligent drug release using alginate as a microcapsule in drug delivery systems [27,29]. A recent study has shown that combining alginate with collagen to form a collagen–alginate hydrogel composite not only improves the mechanical characteristics, such as stiffness and degradation of the material by changing the Ca^2+^ concentration, but it also allows better cell adhesion and attachment [36], cementing the way for future advanced use of alginate in endodontics. Furthermore, the development of new classes of alginate with distinct chemical structures, molecular weights, and cross-linking properties may potentially revolutionise the use of this organic marine biopolymer in endodontics.

The OHAT Risk of Bias Tool is commonly employed to assess the study quality and internal validity of in vitro, in vivo, or ex vivo studies. The advantage of using the OHAT tool is that it includes the domain ‘allocation’, which considers randomization and the idea that every sample should have an equal chance of being assigned to any experimental group. Additionally, it addresses the study’s attrition as well as selective reporting [37]. All the studies included in the present review were classified as Tier 1, indicating that they had excellent internal validity and a high degree of confidence. However, all included studies had either ‘probably high’ or ‘definitely high’ risk of bias for domains 2 and 6. In experimental research, concealed allocation is pivotal as it hides the categorisation of samples into various groups and prevents sample grouping from influencing the outcomes. Allocation concealment may be accomplished in future experimental studies by using sequentially numbered, opaque, sealed envelopes that prevent researchers from seeing the samples used in the experiments [38]. While it is understood that blinding researchers to distinct experimental groups is not always feasible, researchers should integrate alternative methodologic protections and acknowledge the methods’ limitations. 

Although the OHAT tool is well-known for allowing RoB comparisons across a body of evidence and facilitating data comparisons from several evidence streams, the use of this assessment tool alone as a metric of data quality is one of the current review’s shortcomings. It should be highlighted that internal validity (RoB), external validity (directness), and completeness in reporting are all key elements in evaluating a research’s credibility [21]. Another limitation is that only research articles published in English are included, which may introduce a language bias and lead to erroneous interpretations. However, the current review offers valuable preliminary information about the potential of incorporating alginate in the field of endodontics and opens the possibility for further research.

## 5. Conclusions

The present review thoroughly identifies, summarises, and appraises the uses of alginate as a potentially venerable biomaterial in various endodontic applications. Considering the limited available evidence, alginate has emerged as a new biomaterial as a cell carrier and scaffold in regenerative endodontics, a microcapsule delivery system for intracanal medicaments, a chelating agent reinforcing material, and a root canal sealer. Inevitably, one of the major aspirations in the coming decade is the advent of alginate-based endodontic materials that may be used clinically with promising results. Thus, more well-designed experimental research and clinical trials are needed to accomplish this goal by elucidating the efficacy of marine-derived alginate-based biomaterials, which has a substantial impact in the field of dental sciences, particularly endodontics.

## Figures and Tables

**Table 1:** Characteristics of the included studies.

Author	Year	Country	Study Design	Theme	General Outcome
Zhang R et al. [22]	2020	China	In vitro and in vivo	Endodontic regeneration	hDPSCs and vascular endothelial growth factor (VEGF) were co-encapsulated in injectable hybrid RGD-alginate/laponite (RGD-Alg/Lap) hydrogel microspheres, which demonstrated adequate rheological properties, degradation rate, and cell viability. Additionally, it was found to promote the regeneration of pulp-like tissues and generate new microvessels.
Evelyna A et al. [23]	2019	Indonesia	In vitro	Intracanal medicament	When nanocellulose is combined with alginate and subsequently loaded with CHX digluconate 2% (*w*/*v*), the microcapsule appeared to be a viable option for intracanal drug delivery at pH 5.5 and pH 7.5.
Devillard R et al. [13]	2016	France	In vitro and ex vivo	Endodontic regeneration	When compared to synthetic materials, the collagen–alginate composite scaffold may offer significant advantage by allowing a favourable root canal healing environment amenable to regenerative endodontics.
Matsumoto N et al. [24]	2014	Japan	In vivo and in vitro	Endodontic regeneration	The findings showed that EMD does not irritate periapical tissue and may generate a favourable environment for periapical tissue recovery in comparison to PGA.
Girard S et al. [25]	2005	Switzerland	In vitro	Chemical preparation (Chelatingagent)	Aqueous gel containing 1-hydroxyethylidene-1, 1-bisphosphonate (HEBP) with 2% alginate appeared advantageous as a chelating agent over currently available product.
Lambricht L et al. [26]	2014	Belgium	In vitro and in vivo	Endodontic regeneration	Commercially available hyaluronic acid-based formulation can be a suitable delivery system for SCAP-based dental pulp regeneration strategies.
Nurdin D et al. [27]	2013	Indonesia	In vitro	Intracanal medicament	Silica microcapsules coated with sodium alginate and chitosan may be a promising carrier for releasing 2% CHX in the root canal at pH 6.5, as opposed to the normal pH of 7.4.
Athirasala A et al. [14]	2018	USA	In vitro	Endodontic regeneration	The suggested new bioink with alginate hydrogel demonstrated cytocompatibility and natural odontogenic potential, and it can be employed to manufacture scaffolds with sophisticated three-dimensional microarchitectures in the future for regenerative dentistry.
Bhoj M et al. [5]	2015	Hong Kong, China	In vitro	Endodontic regeneration	Simple templating allows RGD-alginate scaffolds to be constructed. When dual growth factors were added to cocultures of stem cells within RGD-alginate scaffolds, microenvironments were created that dramatically enhanced the proliferation of dental pulp stem cell/human umbilical vein endothelial cell combinations.
Huang G et al. [6]	2021	China	In vitro	Endodontic filling materials	The novel algin incorporated BG-based sealer exhibited acceptable flow, film thickness, setting time, solubility, and radiopacity with no cytotoxic effects on MG-63 cells. Dense hydroxyapatite crystals were found on the surface after 4 weeks of immersion in SBF. Furthermore, no difference in sealing performance was noted when compared to commercialised bioceramic sealer.
Yu H et al. [12]	2019	China	In vitro	Endodontic regeneration	The 3D-printed Alg-Gel scaffold is more suitable for the proliferation of hDPSCs than the Alg-Gel scaffold, and the scaffold extracts can better enhance cell proliferation and differentiation.
Liang X et al. [28]	2022	China	In vitro and in vivo	Endodontic regeneration	GelMA-alginate core-shell microcapsule system for co-cultivating and delivering hDPSC and HUVEC without microcapsule aggregation. The microcapsule system enhances cell proliferation, shows greater osteo- and odontogenic, and vasculogenic capacity.
Lai WY et al. [29]	2021	Taiwan, Republic of China	In vitro	Endodontic regeneration	hDPSC-based cell blocks with alginate–fish gelatine hydrogel core and Si ion-infused fish gelatine methacrylate hydrogel shell surrounding HUVEC were able to facilitate regeneration. The capacity to release Si ions improved numerous angiogenic signalling, increased the expression and secretion of angiogenesis-related and odontogenic-related biomarkers.

RGD: arginine–glycine–aspartate; CHX: chlorhexidine; EMD: emdogain; PGA: propyl glycol alginate; SCAP: stem cells from the apical papilla; BG: bioactive glass; SBF: simulated body fluid; Alg-Gel: alginate–gelatine; hDPSC: human dental pulp stem cell; GelMA: gelatine methacryloyl; hDPSC: human dental pulp stem cell; HUVEC: human umbilical vein endothelial cell; Si: silicone.

**Table 2:** Risk of bias of the included studies based on OHAT risk of bias assessment tool.

Studies	Domains									Overall RoB
	1	2	5	6	7	8	9	10	11	
	Was the Administered dose/Exposure Level Adequately Randomized?	Was Allocation to Study Group Adequately Concealed?	Were Experimental Conditions Identical across Study Groups?	Were Research Personnel Blinded to the Study Group during the Study?	Were Outcome Data Complete without Attrition/Exclusion from Analysis?	Can We Be Confident in the Exposure Characterization?	Can We Be Confident with the Outcome Assessment (Including Blinding of Assessors)?	Were All Measured Outcomes Reported?	Were There No Other Potential Threats to Internal Validity?	
Zhang R et al. [22]	PL	PH	DL	DH	PL	DL	DL	DL	DL	Tier 1
Evelyna A et al. [23]	DH	DH	PL	DH	PL	DL	PL	DL	DL	Tier 1
Devillard R et al. [13]	DH	PH	DL	PH	PL	DL	DL	DL	DL	Tier 1
Matsumoto N et al. [24]	PL	PH	DL	PH	PL	DL	DL	DL	DL	Tier 1
Girard S et al. [25]	PL	PH	DL	PH	PL	DL	DL	PL	DL	Tier 1
Lambricht L et al. [26]	PH	PH	PL	DH	PL	PL	PL	PL	DL	Tier 1
Nurdin D et al. [27]	PH	PH	PL	PH	DL	PL	PL	DL	DL	Tier 1
Athirasala A et al. [14]	DH	PH	DL	PH	PL	DL	PL	PL	DL	Tier 1
Bhoj M et al. [5]	DH	DH	DL	DH	PL	DL	DL	DL	DL	Tier 1
Huang G et al. [6]	DH	DH	DL	DH	PL	PL	DL	DL	DL	Tier 1
Yu H et al. [12]	DH	DH	DL	PH	PL	DL	PL	DL	DL	Tier 1
Liang X et al. [28]	DH	DH	DL	DH	PL	DL	DL	DL	DL	Tier 1
Lai WY et al. [29]	PH	PH	DL	PH	DL	DL	PL	DL	DL	Tier 1

DL: Definitely Low; PL: Probably Low; PH: Probably High; DH: Definitely High.

## Data Availability

Not applicable.
